# Incidence and Causes of Reintubation Other Than Reopening of the Chest in Post-Cardiac Surgical Patients in a Tertiary Care Hospital

**DOI:** 10.7759/cureus.14939

**Published:** 2021-05-10

**Authors:** Imtiaz Ahmad, Bahauddin Khan, Mujahid Ul islam, Azam Jan, Omer Farooq, Waasay Hassan Khan, Usman Ghani

**Affiliations:** 1 Anesthesiology, Rehman Medical Institute, Peshawar, PAK; 2 Cardiothoracic Surgery, Rehman Medical Institute, Peshawar, PAK; 3 Surgery, Hayatabad Medical Complex, Peshawar, PAK

**Keywords:** endotracheal reintubation, cardiac surgery, cardiac intensive care unit, cicu, coronary artery bypass grafting (cabg)

## Abstract

Objective

To determine the incidence of endotracheal reintubation, excluding surgical reopening, in post-cardiac surgical patients in a tertiary care hospital.

Material and methods

A retrospective descriptive analysis of 408 patients who underwent different cardiac surgeries during this period. Post-operative extubation was performed when patients fulfilled the preset criteria for extubation, which include consciousness (awake and aware), stable vital signs, acceptable arterial blood gases, acceptable respiratory mechanics, a maximum inspiratory force greater than 20-25 cm H_2_O, chest tube drainage less than 100 ml per hour, normal temperature and electrolytes. The total number of patients who were reintubated within 72 hours of extubation was noted. The criteria for reintubation included altered conscious level with Glasgow Coma Score (GCS) of less than 8, respiratory failure, unstable hemodynamics, and arrhythmias such as ventricular tachycardia (VT) and fibrillation. All of the information was collected retrospectively on a specifically prepared form. Data was entered and evaluated in Statistical Package for the Social Sciences.

The research was piloted in the Cardiac Intensive Care Unit (CICU) of Northwest General Hospital and Research Center, Hayatabad, Peshawar from December 2018 to March 2020.

Results

Out of 408 patients who had cardiac surgeries, only nine (2.2%) were reintubated after initial extubation. The average time for which patients remained on the ventilator was 8 ± 2 hours. The reasons for reintubation were recorded. Among those reintubated, eight patients (88.88%) had undergone coronary artery bypass grafting (CABG) whereas one patient (11.11%) had undergone mitral valve replacement (MVR). In three (33.33%) patients, stroke (hemiplegia in two and global brain ischemia in one) with low GCS was the primary cause of reintubation. Arrhythmias - which included VT, ventricular fibrillation (VF), and supraventricular tachyarrhythmias (SVT) - were responsible for three (33.33%) cases of reintubation. Respiratory failure - with a partial pressure of oxygen in arterial blood less than 60 mmHg, along with tachypnea - was responsible for reintubation in two (22.22%) patients. In one (11.11%) patient who had MVR, cardiac arrest was the underlying reason; the cause of arrest could not be retrieved from the retrospective data. Notably, as a common variable, five (62.5%) out of the eight reintubated CABG patients had a poor left ventricular function.

Conclusion

Causes of reintubation were primarily cardiac (arrhythmias) and neurological, followed by respiratory causes in our center. Patients with poor left ventricular function and diffuse coronary artery disease appear to have a higher incidence of reintubation which can lead to extended CICU and hospital stay, elevated mortality, and higher costs.

## Introduction

Despite the significant improvement seen in cardiac surgical techniques and perioperative care in recent years, postoperative endotracheal reintubation is not uncommon for seriously ailing patients in the cardiac intensive care unit (CICU) after successful weaning from the ventilator [1]. Research describes the reintubation incidence, subsequent to planned extubation, to be in the range of 10-20% in the general intensive care unit (ICU) patients [2]. Literature reports that unsuccessful endotracheal extubation and reintubation are linked with worse outcomes, describing ICU mortality rates of 25-50% among these patients [3]. Regarding post-cardiac surgical patients, literature has shown that the frequency of reintubation, in patients who were previously successfully liberated from mechanical ventilation, is 6.6% [4]. It has been widely established that reintubation in open-heart surgery patients is associated with an elevated incidence of morbidity and mortality; moreover, the length of stay in the ICU and hospital increases leading to elevated expenses and resource utilization [5].

We performed a retrospective descriptive research on patients who were admitted to CICU after various cardiac surgeries and who remained on mechanical ventilation until they fulfilled the preset weaning criteria from positive pressure ventilation [6]. We recorded the number of all those patients who were reintubated and also the type of surgeries and other factors associated with the reinsertion of an endotracheal tube.

## Materials and methods

The research was performed in CICU of Northwest General Hospital and Research Center, Hayatabad, Peshawar, which is a tertiary care hospital. All patients who were effectively extubated in CICU after open-heart surgery, including but not limited to coronary artery bypass grafting (CABG), valve replacement/repair, atrial or ventricle septal defects, and combined procedures, were incorporated in the research. The study began with approval from Hospital Ethical Review Committee and included data for patients from December 2018 to March 2020.

A total of 408 patients were admitted to the CICU after cardiac surgery during this period. Upon data review, we assembled all those patients who were reintubated during this time period and documented the causes of reintubation, patient characteristics, any comorbidities, and the type of surgery they underwent. The data was entered into and analyzed using Statistical Packages for the Social Sciences. The reason for reinsertion of the endotracheal tube was the primary study variable. Incidence and percentage were computed for qualitative variables.

## Results

Demographic statistics revealed a mean age of 57 years. A total of 304 male (74.5%) and 104 female (25.5%) patients comprised the study sample. Most of the patients were reintubated within the first 24 hours of extubation. Table 1 illustrates the demographics and the types of surgeries for all patients included in the study. The majority of patients in the study sample underwent CABG (n = 354; 86.7%); most of these were male patients undergoing CABG (n = 273; 77%).

**Table 1:** Total number of patients distributed by gender and procedure (n = 408). ASD: Atrial septal defect; AVR: Aortic valve replacement; CABG: Coronary artery bypass grafting; DVR: Double valve replacement; MVR: Mitral valve replacement; TC: Total correction; TOF: Tetralogy of Fallot; VSD: Ventricular septal defect.

Gender	Total number of patients	Percentage
Male	304	74.5
Female	104	24.5
Total	408	100
Frequency of patients distributed by procedure		
Procedure	Number of patients	Percentage
CABG	354	86.7%
CABG + MVR	2	0.5%
CABG + AVR	4	1%
MVR	16	4%
AVR	15	3.7%
DVR	3	0.7%
VSD	2	0.5%
ASD	7	1.7%
TOF + TC	2	0.5%
Atrial myxoma	1	0.25%
Aortic root replacement	2	0.5%
Total	408	100%

A total of nine (2.2%) patients were reintubated. Patients undergoing CABG had the highest incidence of endotracheal reintubation, with CABG preceding 8/9 (88.88%) reintubations. This was followed by MVR which was responsible for 1/9 (11.11 %) reintubations (Table 2).

**Table 2:** Frequency of reintubated patients with respect to procedures. CABG: Coronary artery bypass grafting; MVR: Mitral valve replacement.

Procedure	Frequency	Number of reintubations	Percentage
CABG	354	8	2.25%
MVR	16	1	6.25%
Other procedures	38	0	0
Total	408	9	2.21%

Table 3 dissects further the characteristics of the reintubated patients with regards to gender distribution. Male patients, predominantly those undergoing CABG, were the leading demographic among those who eventually required reintubation.

**Table 3:** Characteristics of reintubated patients with respect to gender and procedure. CABG: Coronary artery bypass grafting; MVR: Mitral valve replacement.

Gender	Reintubated	CABG	MVR
Male (n = 304)	8 (2.63%)	7	1
Female (n = 104)	1 (0.96%)	1	0
Total (n = 408)	9 (2.21%)	8	1

The underlying reasons for endotracheal reintubation were diverse, as demonstrated in Table 4. The leading causes were stroke and arrhythmias, each responsible for three patients (33.3% each) requiring reintubation. Respiratory complications lead to reintubation in two patients (22.22%). Finally, one patient (11.11%) was reintubated for cardiac arrest. Interestingly, poor left ventricular function was a common variable in five (62.5%) out of the eight reintubated CABG patients.

**Table 4:** Causes of reintubation.

Cause	Frequency	Percentage
Arrhythmias	3	33.33%
Stroke	3	33.33%
Respiratory	2	22.22%
Cardiac arrest	1	11.11%

## Discussion

Re-intubation after successful initial extubation is not uncommon after cardiac surgery, with frequency rates reported to be around 1.1%-6.6% in previous studies [5-9]. One observational research done on cardiac surgery patients found the reintubation frequency to be 2.14%, which is analogous to our research [10]. Reintubation after cardiac surgery is multifactorial involving cardiovascular, respiratory, and central nervous system causes. One of the more common complications post-cardiac surgery that may warrant reintubation is the development of pleural effusion. Studies have shown pleural effusions to be abundantly prevalent following cardiac surgery, requiring reintubation in a significant number of patients [11]. 

In our study, the overwhelming majority of patients who required reintubation were patients who had undergone CABG as the primary surgical procedure. This is widely reflected in the current literature which describes a higher frequency of reintubation in CABG patients, along with those who undergo MVR surgery [2]. Additionally, the frequency of reintubation in our study was higher in male patients (2.63%) as compared to female patients (0.96%). The higher prevalence of diabetes mellitus in males as compared to female patients presumably was a contributory factor to this finding. 

Among cardiovascular causes, arrhythmias (ventricular tachycardia and supraventricular tachycardia) have been widely regarded as a recurring indication for reintubation in cardiac surgery patients; this is especially important because they are one of the major causes of morbidity and mortality [12-15]. In our research study, arrhythmias with hemodynamic compromise were expectedly responsible for a significant number of reintubations (33.3% of total reintubations). A similar number of patients (33.3%) required reintubation for neurological causes, specifically cerebrovascular accidents (stroke with hemiplegia in two cases and global brain ischemia in one patient). It is widely known that patients undergoing CABG surgeries, along with valvular surgeries, are at an elevated risk of neurological complications, primarily due to the risk of cerebral embolization [16,17].

The third leading cause of reintubation, which accounted for 22.22% of cases, were respiratory issues with impending respiratory failure evident by amplified work of breathing in 50% of these cases. The relatively low incidence of respiratory issues may be attributed to our practice of using short-acting opioid fentanyl intraoperatively (in a dose of 12-15 microgram per kg) and the use of propofol as intravenous anesthetic induction agent as well as isoflurane as anesthetic maintenance agent; this is followed by the use of nonopioid analgesics like ketorolac, intravenous acetaminophen, and nalbuphine in CICU. This frequency of reintubation for respiratory issues is comparable to rates seen in other studies reported in the literature [2,10]. Indications for reintubation, including pleural effusions as previously mentioned, are a leading cause of prolonged ICU stay in patients undergoing a variety of thoracic surgical procedures. Lastly, one patient (11.11%) required reinsertion of an endotracheal tube as a part of resuscitation efforts secondary to cardiac arrest due to VT.

With regards to outcomes of the reintubated patients, three patients who were reintubated due to cardiac arrhythmias (VT, SVT) recovered after 8-10 hours of reintubation; they were subsequently extubated and discharged from the ICU. The three patients who were reintubated secondary to stroke (hemiplegia, global brain ischemia) had tracheostomy after 24 hours of ventilation, were weaned from the ventilator to oxygen mask in 3-5 days, and were transferred to the tracheostomy care unit. Two patients that suffered from respiratory failure also recovered within 6-8 hours of ventilation. Finally, the patient reintubated due to cardiac arrest died six hours after reintubation. 

The results of our study broadly mirror those seen in other related research studies evaluating reintubation and its indications in post-cardiac surgery patients. The incidence of ventilator-associated pneumonia was higher in patients that were reintubated for strokes as the ventilation time was longer for these patients compared to the rest. Lack of follow-up data for the reintubated patients regarding their discharge from the hospital meant that we could not evaluate the total length of stay in the hospital for each patient.

## Conclusions

The predominant reasons for reintubation after cardiac surgery are arrhythmias with hemodynamic instability, neurological and respiratory complications. Patients undergoing cardiac surgery with poor cardiac reserve or function carry a higher risk of reintubation. Reintubation prolongs ventilation, CICU, and hospital stay, thus increasing morbidity, mortality, and cost.

We recommend that further studies be conducted in post-cardiac surgery patients to clearly recognize additional factors and circumstances that could predict a decline in the clinical state. Timely management of the risk factors involved will lead to a reduction in reintubation rates and improved clinical outcomes.
